# Variants in SNCA Gene Are Associated with Parkinson’s Disease Risk and Cognitive Symptoms in a Brazilian Sample

**DOI:** 10.3389/fnagi.2017.00198

**Published:** 2017-06-20

**Authors:** Clarissa L.C Campêlo, Fernanda C. Cagni, Diego de Siqueira Figueredo, Luiz G. Oliveira Jr., Antônio B. Silva-Neto, Priscila T. Macêdo, José R. Santos, Geison S. Izídio, Alessandra M. Ribeiro, Tiago G. de Andrade, Clécio de Oliveira Godeiro, Regina H. Silva

**Affiliations:** ^1^Memory Studies Laboratory, Physiology Department, Universidade Federal do Rio Grande do NorteNatal, Brazil; ^2^Molecular Biology and Gene Expression Laboratory, Universidade Federal de AlagoasArapiraca, Brazil; ^3^Medicine Department, Universidade Federal do Rio Grande do NorteNatal, Brazil; ^4^Bioscience Department, Universidade Federal de SergipeItabaiana, Brazil; ^5^Department of Cell Biology, Embryology and Genetics, Universidade Federal de Santa CatarinaFlorianópolis, Brazil; ^6^Biosciences Department, Universidade Federal de São PauloSantos, Brazil; ^7^Faculty of Medicine, Universidade Federal de AlagoasMaceió, Brazil; ^8^Behavioral Neuroscience Laboratory, Pharmacology Department, Universidade Federal de São PauloSão Paulo, Brazil

**Keywords:** Parkinson’s disease, alpha-synuclein, SNCA gene, polymorphism, cognitive impairment, clinical assessment, Brazil

## Abstract

Genetic susceptibility contributes to the etiology of sporadic Parkinson’s Disease (PD) and worldwide studies have found positive associations of polymorphisms in the alpha-synuclein gene (SNCA) with the risk for PD. However, little is known about the influence of variants of SNCA in individual traits or phenotypical aspects of PD. Further, there is a lack of studies with Latin-American samples. We evaluated the association between SNCA single nucleotide polymorphisms (single nucleotide polymorphisms, SNPs – rs2583988, rs356219, rs2736990, and rs11931074) and PD risk in a Brazilians sample. In addition, we investigated their potential interactions with environmental factors and specific clinical outcomes (motor and cognitive impairments, depression, and anxiety). A total of 105 PD patients and 101 controls participated in the study. Single locus analysis showed that the risk allele of all SNPs were more frequent in PD patients (*p* < 0.05), and the associations of SNPs rs2583988, rs356219, and rs2736990 with increased PD risk were confirmed. Further, the G-rs356219 and C-rs2736990 alleles were associated with early onset PD. T-rs2583988, G-rs356219 and C-2736990 alleles were significantly more frequent in PD patients with cognitive impairments than controls in this condition. In addition, in a logistic regression model, we found an association of cognitive impairment with PD, and the practice of cognitive activity and smoking habits had a protective effect. This study shows for the first time an association of SNCA polymorphism and PD in a South-American sample. In addition, we found an interaction between SNP rs356219 and a specific clinical outcome, i.e., the increased risk for cognitive impairment in PD patients.

## Introduction

Parkinson’s disease (PD) is the second most common neurodegenerative disease. This condition mainly affects motor function, but also causes non-motor symptoms (Fahn, 2003; Wirdefeldt et al., 2011; Pihlstrøm et al., 2013). Among other neurological sings, the cardinal features of this disorder are bradykinesia, rigidity and resting tremor. In parallel, impairment of executive functions and the presence of apathy, anxiety, and depression are the main neuropsychiatric manifestations in PD patients (Rodriguez-Oroz et al., 2009). The onset of PD is usually after 50 years old, and a sharp increase of the incidence is seen after the age of 60 (1% of the population; Lau and Breteler, 2006). Although PD’s etiology remains unclear, the interaction between genetic and environmental substrates has been associated with the development of the disease (Lau and Breteler, 2006; Wirdefeldt et al., 2011). Among those environmental factors, several studies pointed the inverse correlation between cigarette smoking and PD risk (Allam et al., 2004; Li et al., 2015). On the other hand, history of professional pesticide exposure, rural living or well water drinking were reported to increase PD risk (Semchuk et al., 1991; Firestone et al., 2005). In addition, physical activity (Paillard et al., 2015; Shih et al., 2016), cognitive reserve (Hindle et al., 2014, 2015) and caffeine intake (Costa et al., 2010) are suggested as protective factors, but with insufficiently consistent results.

Genome-wide association studies (GWAS) have identified single nucleotide polymorphisms (SNP) in many candidate genes that contribute to PD susceptibility such as microtubule-associated protein tau (MAPT), leucine-rich repeat kinase (LRRK2) and alpha-synuclein (SNCA) (Mata et al., 2011; Satake et al., 2009; Simón-Sánchez et al., 2009; Sharma et al., 2012). The relevance of SNCA variations for PD risk is already well established through linkage and GWAS studies. Moreover, certain polymorphisms of SNCA are among the major risk factors for sporadic PD (Simón-Sánchez et al., 2009) and have been correlated with increased plasmatic levels of alpha-synuclein (Mata et al., 2010).

The presynaptic protein alpha-synuclein is the major component of the Lewy body, which is the pathological hallmark of PD (Spillantini et al., 1997, 1998; Trojanowski and Lee, 1998; Xu et al., 2015). The physiological function of alpha-synuclein implicates molecular mechanisms of dopaminergic neurotransmission such as regulation of oxidative stress, maintenance of synaptic function and neuronal trafficking (Schapira, 2007; Bendor et al., 2013; Eisbach and Outeiro, 2013). The overexpression of alpha-synuclein reduces tyrosine hydroxylase activity and dopamine release (Perez et al., 2002; Ozansoy and Basak, 2012), disrupts microtubule-dependent trafficking (Lee et al., 2006), increases oxidative by complex I mitochondrial dysfunction (Mullin and Schapira, 2013; Wu-Chou et al., 2013) and impairs neurotransmitter storage which leads to cytoplasmic accumulation (Lotharius and Brundin, 2002). Mutant protein can result in increased dopamine intracytoplasmic concentration, which contributes to raise the sensitivity to dopamine toxicity by reactive oxygen species generation (Tabrizi et al., 2000).

Case-control studies in different populations have also found associations between several SNCA polymorphisms and increased risk of PD. For example, the dinucleotide repeat REP1 located in SNCA promoter (SNCA-Rep1) and the 3′ untranslated region (UTR) variants have been broadly investigated (Pals et al., 2004; Hu et al., 2010; Ritz et al., 2012). Variations in these regions may confer susceptibility to PD by altering transcription factor binding sites (Chiba-Falek and Nussbaum, 2001; Chiba-Falek et al., 2003) and generating or destroying microRNA target sites, which in turn modifies gene expression (Wang et al., 2008; Sotiriou et al., 2009; Mccarthy et al., 2011). Several investigations had focused on the association between SNCA SNPs and PD in ethnic groups, most of them performed in Caucasian and Asian populations. Hence, the results may be applicable only for these groups (Han et al., 2015). To our knowledge, no studies have investigated associations of SNCA polymorphisms in South American populations.

Currently, there is much interest in the search for clinical predictors of motor and non-motor symptoms in PD. Notwithstanding, most of the gene association studies are limited to genetic risk factor data. Importantly, the consequences of genetic variability on clinicalphenotypes, as well the interaction between genetic and environmental substrates, are poorly elucidated. Few studies pointed weak or absence of associations with clinical outcomes such as motor impairment (Ritz et al., 2012; Markopoulou et al., 2014), anxiety, or depression (Verbaan et al., 2008; Guo et al., 2014; Chen W. et al., 2015; Cheng et al., 2016), sleep, and autonomic disorders (Verbaan et al., 2008; Chen W. et al., 2015), and cognitive impairments (Verbaan et al., 2008; Guo et al., 2014; Chen W. et al., 2015; Chen Y.P. et al., 2015; Cheng et al., 2016; Wang et al., 2016). Thus, there is little information concerning motor and non-motor symptoms assessment, lifestyle and environmental expositions. Gene-environmental studies investigate whether environmental factors such as smoking habits and coffee consumption could modify genetic associations with PD (Gao et al., 2012; Miyake et al., 2012; Ritz et al., 2012; Trotta et al., 2012). However, studies that investigate possible SNCA polymorphisms associations with specific clinical aspects of PD are inconclusive. Therefore, the elucidation of the genetic contribution to clinical phenotypes remains a challenge. In this respect, the description of genetic predictors and their relationship with other etiological factors and clinical outcomes is determinant to improve the knowledge of pathophysiological pathways and help to target the best therapeutic program.

In the present study, we investigated possible interactions between polymorphisms in the SNCA gene and PD in a Brazilian sample, and examined potential associations between these polymorphisms and environmental factors and specific clinical outcomes.

## Subjects And Methods

### Patients and Controls

The unrelated sample consisted of 105 PD patients and 101 control subjects recruited from Onofre Lopes University Hospital, in Rio Grande do Norte (Northeastern – Brazil) from June, 2013 to November, 2014. PD was diagnosed by a neurologist according to UK Parkinson’s Disease Society Brain Bank Clinical Criteria (Hughes, 2004). Recruitment of control subjects was conducted in the same hospital at other departments than the neurology department. Controls were subjects from the general population without neurological disease and family history of PD. The groups were matched by age and sex. This study was approved by the ethical committee of Onofre Lopes University Hospital (protocol number 04261012.5.1001.5292). All the patients and controls were requested to sign the written informed consent.

### Clinical Assessment

Case and control subjects filled out a set of eight questionnaires. Information of demographic variables and medical history, such as sex, age, education level, age at PD onset and disease duration were obtained in the baseline interview. Family history was considered positive until second-degree relatives. A structured questionnaire of environmental factors delivered information about risk (pesticide exposition, living in rural areas and well-water consumption) and protective (smoking habits, coffee intake, physical exercises and cognitive activities) factors. History of smoking was defined on basis of self-report as never vs. ever having smoked at least once a day for at least one year (Miyake et al., 2012). Coffee consumption was assessed on basis of self-report and consumption was defined as more than two coffee cups per day (Trotta et al., 2012). Similarly, self-reported physical exercises or cognitive actives (i.e., reading, crossword puzzles, card games, chess, and others) were considered when carried out at least once a week.

The evaluation of different clinical aspects comprised the application of the following inventories: (1) PD motor symptoms were assessed by Unified Parkinson’s Disease Rating Scale (UPDRS I, II, and III) (Fahn and Elton, 1987), Hoehn & Yahr scale (HY) (Hoehn and Yahr, 1967) and Schwab and England Activities Daily Living Scale (SE); (2) emotional status was assessed by Beck Depression Inventory (BDI) (Beck et al., 1988b) with a cut-off score of 10 to detect depression (Tröster et al., 1995) and Beck Anxiety Inventory (BAI) (Beck et al., 1988a) with a cut-off score of 10 to detect anxiety (Julian, 2011). The severities of depression and anxiety were determined, according to the following scores, respectively: moderate (BDI: 19–29 points; BAI: 20–30 points) and severe (BDI: higher than 29 points; BAI: higher than 30 points); (3) Mini Mental State Examination (Folstein et al., 1975) and Frontal Assessment Battery (FAB) (Beato et al., 2007) assessed cognitive functions. Cognitive impairment was defined by the application of the cut- off MMSE scores taking the educational level into consideration: 20 for illiterate, 25 for lower education (1 to 4 years), 27 for middle education (5 to 8 years), and 28 for high education (greater than 8 years) (Brucki et al., 2003). All clinical evaluations were conducted during the “on” state of levodopa treatment.

### DNA Extraction and Genotyping

Genomic DNA was extracted from EDTA-containing peripheral blood samples (commercial kit FlexiGene^®^ DNA kit, Qiagen, Germany). Genotyping of SNPs rs2583988 (C > T), rs356219 (A > G), rs2736990 (T > C), and rs11931074 (G > T) in SNCA gene was performed using real-time TaqMan^®^ polymerase chain reaction assay according to the manufacturer’s instructions (Applied Biosystems, Foster City, CA, United States). Four samples were lost due to poor quality of DNA. 104 PD patients and 98 controls were successfully genotyped.

### Statistical Analysis

Non-parametric data were presented as median [minimum value; maximum value]. Inventories scores were compared between groups with Mann-Whitney and Kruskal-Wallis given the nonparametric nature of the data. *X*^2^ statistics (Fisher exact test) was used to compare categorical data, calculation of frequency significance and odds ratio (OR). Parametric data were presented as mean and standard deviation. *T*-student independent test was used to assess differences in mean age at interview between PD and control groups. Calculations of Hardy–Weinberg equilibrium, linkage disequilibrium (LD), estimation of haplotypes and haplotype frequency were performed by the software Snpstat^1^. Crude odds ratios (ORs) and 95% confidence intervals (CIs) were calculated to estimate risk size for the heterozygotes and homozygotes for the risk alleles by binary logistic regression analysis, adjusted for age and gender. The statistical power calculation was performed using G ^∗^ Power statistical program. Statistical significance was defined when *p*-values < 0.05.

## Results

### Study Population

Two hundred and six subjects participated in the study. In PD group (*n* = 105, 73 men and 32 women) the mean age was 64.42 years (range: 37–89 years) and in control group (*n* = 101, 68 men and 33 women) it was 62.98 years (range: 38–88 years), without significant difference (*U* = 4632.00, *p* = 0.260) (**Table 1**). The majority of PD and control subjects were married, living in urban area and had basic education (35.7% of cases and 35.3% of controls had less than four years of education). There were no differences in percent of subjects in each level of education between groups (*p* = 0.486). Most of PD cases (81.4%) were retired while 49% of controls subjects had a work activity.

**Table 1:** Profile and frequencies of exposition to environmental factors.

	Cases (*n* = 105)	Control (*n* = 101)	Unadjusted OR (95% CI)	*p*
**Mean age at interview (years)^a^**	64.42 ± 11.69	62.98 ± 10.04	–	0.259
**Mean onset age (years)^a^**	55.7 ± 11.9	–	–	–
**Mean disease duration**	8.80 ± 5.78	–	–	–
**Familial history (%)**	42	–	–	–
**Early onset (%)^b^**	35.2	–	–	–
**Education (%)**				
Illiterate	11.9	17.2	–	0.486
Low^c^	35.7	35.3		
Midlle^d^	15.8	11.1		
Higher^e^	36.6	36.4		
**Risk Factors**				
**Pesticides (%)**				
No	77.2	73.0	0.79 (0.42–1.51)	0.511
Yes	22.8	27.0		
**Countryside (%)**				
No	37.3	40.0	1.12 (0.63–1.97)	0.773
Yes	62.7	60.0		
**Well-water consumption**				
No	27.5	25.0	0.88 (0.47–1.65)	0.750
Yes	72.5	75.0		
**Protective Factors**				
**Coffee consumption (%)**				
No	71.7	81.4	0.58 (0.29–1.27)	0.106
Yes	28.3	18.6		
**History of Smoking (%)**				
No	55.4	51.5	0.85 (0.49–1.48)	0.671
Yes	11.9	20.2		
Abstinent	32.7	28.3		
**Physical Activity (%)**				
No	54.4	68.0	1.64 (0.92–2.91)	0.110
Yes	43.6	32.0		
**Cognitive Activity (%)**				
No	45.5	17.3	**0.25 (0.13–0.48)**	**<0.001**
Yes	54.5	82.7		

^a^*Data presented as mean ± standard deviation. Groups compared by independent sample *t*-test*.

^b^*≤50 years*

^c^*Less than 4 years of education*

^d^*Between 4 and 8 years of education*

^e^*9 years of education or more*

*p-value, odds ratios and confidence intervals (CI) calculated by Fisher’s test for frequencies of environmental factors. Chi-square test evaluated frequencies of educational level. Significant p-values are indicated in bold*.

### PD Group’s Profile

The mean age of PD onset was 55.7 ± 11.9 years (range 30–87), with 37 patients (35.2%) classified as early onset (≤50 years). The mean of disease duration was 8.80 ± 5.78 years (range 1–32) and of treatment duration was 5.6 ± 4.5 years (range 0–21). Seventy patients (69.3%) describe their first symptom as involuntary tremors. A positive family history was reported by 45 patients (42.9%). Those cases were categorized as “familial”, the remaining subjects as “sporadic” (**Table 1**).

Frequencies of exposition to environmental factors are shown in **Table 1**. We found a similar frequency of exposure to risk factors (pesticide contact, living in countryside and well-water consumption) and protective factors (coffee consumption, smoking habits, and physical activities) between groups, without significant associations with PD risk. Although most of the subjects reported living in countryside (62.7% in cases and 60% in controls), there were few reports of pesticide use in agriculture (22.8% in cases and 27% in controls). There was a low frequency of reported coffee consumption (28.3% in cases and 18.6% in controls) and current smokers (11.9% in cases and 20.2% in controls) in our sample. We found a higher frequency of practice of cognitive activities in the control group, and this factor showed a strong protective effect against PD (OR = 0.25; 95% CI = 0.13–0.48).

### Clinical Assessment

Performances of PD and control group in the clinical assessment are described in **Table 2**. Median of disease progression measured by Hoehn & Yahr scale was 2.5 [1–5], and 53% of the patients were in stages III to V. Assessment of motor activity measured by UDPRS III indicated a significant motor impairment in PD group [19.0 [0–50] versus 0.0 [0–19]; U = 181.0, *p* < 0.001]. PD patients also showed greater difficulty to perform daily living activities (20.0 [3–45 versus 0.0 [0–12]) when evaluated by UDPRS II (*U* = 82.5, *p* < 0.001). In addition, patients presented a reduction of functional independence (70.0 [10–90] versus 100.0 [60–100]; *U* = 296.0, *p* < 0001).

**Table 2:** Clinical assessment scores of Pakinson’s disease (PD) patients and Controls.

	PD	Control	*p*
**Hoehn & Yahr**	2.5 [1;5]	0.0	–
**UPDRS I**	1.0 [0;4]	2.0 [0;11]	**0.006**
**UPDRS II**	20.0 [3;45]	0.0 [0;12]	**<0.001**
**UPDRS III**	19.0 [0;50]	0.0 [0;19]	**<0.001**
**SE**	70.0 [10; 90]	100.0 [60;100]	**<0.001**
**MMSE score**	23.0 [10;30]	25.0 [8.;30]	0.097
**Cognitive impairment (%)^a^**	61.0	57.9	0.663
**MMSE**			
Illiterate	21.0 [10;25]	22.0 [8;29]	0.187
Lower	21.0 [11;27]	25.0 [19;30]	**0.003**
Middle	26.0 [12;29]	25.0 [15;30]	0.904
Higher	26.0 [11;30]	25.0 [13;30]	0.667
**FAB**	11.0 [2;16]	11.0 [2;18]	0.063
Illiterate	6.0 [2;11]	8.0 [4;16]	**0.011**
Lower	7.0 [2;16]	9.0 [4;18]	**0.027**
Middle	13.0 [2;16]	12.0 [2;18]	0.790
Higher	13.0 [2;16]	14.0 [3;18]	0.279
**BDI score**	14.0 [2;47]	5.0 [0;47]	**<0.001**
**Depression (%)^b^**	74.7	29.0	**<0.001**
**BAI score**	16.0 [0;47]	7.0 [0;37]	**<0.001**
**Anxiety (%)^c^**	72.6	36.0	**<0.001**

*PD, Parkinson’s Disease; UPDRS, Unified Parkinson’s Disease Rating Scale; SE, Schwab & England Activities Daily Living Scale; MMSE, Mini Mental State Examination; BDI, Beck Depression Inventory; BAI, Beck Anxiety Inventory*

^a^*Applied cut-off point for education level to MMSE scores (illiterate: 20, lower education:25, middle education: 27, high education: 28)*.

^b,c^*Presence of depression and anxiety after applied cut-off points*.

*Data are reported as median, minimum and maximum values; *p*-value by Mann–Whitney test or chi-square for frequencies. Significant *p*-values are indicated in bold*.

Total score of MMSE (PD: 23[10-30] versus Control: 25.0 [8–30]) and FAB PD: 11.0 [2–16] versus control: 11.0 [2–18]) (**Table 2**), as well as the presence of cognitive impairment (61% in PD and 57.9% in control group) did not differ between groups [χ^2^(1) = 0.243; *p* = 0.663]. However, we observed a significant lower score of MMSE in PD group after the stratification of data by educational levels, which indicates a larger cognitive impairment in the subgroup of PD patients with lower education compared to controls with the same level of education (21.0 [11–27] versus 25.0 [19–30]) (*U* = 336.50, *p* = 0.003). Similar effect of education level was found after the stratification of FAB scores. Regarding emotional evaluation, PD group had significantly higher scores in BDI (11.0 [2–47] versus 5.0 [4–74]) and BAI (16.0 [0–47] versus 7.0 [0–37]) than the control group. Furthermore, levels of depression and anxiety were significantly more severe in the PD group (BDI: 24.2% moderate and 10.5% severe; BAI: 26.3% moderate and 9.5% severe, respectively), which resulted in a higher frequency of depression [74.7% versus 2%; χ^2^(1) = 40.971, *p* < 0.001] and anxiety symptoms [72.6% versus 36.0%; χ^2^(1) = 26.305, *p* < 0.001] in the PD group.

### SNP Assessment

Deviation from Hardy-Weinberg equilibrium was not observed for any of the SNPs. 104 participants in PD group and 98 in control group were genotyped for all SNPs. Linkage disequilibrium (LD) structure of SNCA indicates a significant pairwise value of LD between rs356219 and rs2736990 (*r*^2^ = 0.888, *D*′ = 0.988, *p* < 0.001). Allele and genotype distributions of SNPs in patients and controls are summarized in **Table 3**.

**Table 3:** Genotypic and allelic frequencies of single nucleotide polymorphisms (SNPs) in alpha-synuclein gene (SNCA) gene in PD cases (*n* = 104) and controls (*n* = 98).

Genotype	PD *n*(%)	Control *n*(%)	Unadjusted analysis		Adjusted analysis	
			OR (95% CI)	*p*	OR (95% CI)	*p*
**rs2583988**						
C/C	55(53)	61 (62)	1.00 (reference)		1.00 (reference)	
C/T	38 (37)	36 (37)	1.17 (0.65–2.09)	0.597	1.19 (0.65–2.15)	0.562
T/T	11 (11)	1 (1)	12.20 (1.52–97.58)	**0.018**	12.35 (1.52–99.88)	**0.018**
C allele	148 (71.1)	158 (80.6)	1.68 (1.06–2.68)	**0.026**		
T allele	60 (28.8)	38 (19.3)				
**rs356219**						
A/A	16 (15)	26 (27)	1.00 (reference)		1.00 (reference)	
G/A	50 (48)	51 (52)	1.59 (0.76–3.32)	0.214	1.60 (0.76–3.35)	0.209
G/G	38 (37)	21 (21)	2.94 (1.29–6.67)	**0.010**	2.94 (1.29–6.72)	**0.010**
A allele	82 (39.4)	103 (52.5)	1.70 (1.14–2.52)	**0.008**		
G allele	126 (60.5)	93 (47.4)				
**rs2736990**						
T/T	14 (13)	19 (19)	1.00 (reference)		1.00 (reference)	
T/C	43 (41)	55 (56)	1.06 (0.47–2.35)	0.884	1.18 (0.52–2.66)	0.687
C/C	47 (45)	24 (24)	2.65 (1.13–6.20)	**0.024**	2.73 (1.15–6.48)	**0.022**
T allele	71 (34.1)	93 (47.4)	1.74 (1.16–2.60)	**0.006**		
C allele	137 (65.8)	103 (52.5)				
**rs11931074**						
G/G	54 (52)	61 (62)	1.00 (reference)		1.00 (reference)	
G/T	39 (38)	29 (30)	1.51 (0.83–2.78)	0.175	1.52 (0.83–2.80)	1.70
T/T	11 (11)	8 (8)	1.55 (0.58–1.14)	0.379	1.56 (0.58–4.18)	0.377
G allele	146 (70.1)	151 (77.0)	1.40 (0.89–2.19)	0.138		
T allele	61 (29.3)	45 (22.9)				

*PD, Parkinson’s disease; OR, odds ratio; CI, confidence interval; *p*, significance level*.

*Significant p-values are indicated in bold*.

Single locus analysis of SNPs rs2583988, rs356219, rs2736990, and rs11931074 showed that the risk alleles of each SNP, as well as homozygotes for these alleles, were more frequent in PD patients compared to controls. Logistic regression analysis confirmed a significant association between risk genotypes and PD for rs2583988 (OR = 12.20, 95%IC: 1.52–97.58, *p* = 0.018), rs356219 (OR = 2.94, 95%IC: 1.29–6.67, *p* = 0.010), rs2736990 (OR = 2.65, 95%IC: 1.16–2.60, *p* = 0.024), that remained significant after correction for the covariates age and sex. There was no significant difference in genotype distribution for rs11931074 between patients and controls (OR = 1.55, 95% IC: 0.58–1.14, *p* = 0.379). Statistical power calculation applied to the sample size used in this research indicate a detection of a gene-disease association for SNPs rs2583988, rs356219, rs2736990 with values of OR higher than 2.60, with an accuracy between 68 and 85% under the recessive model.

Age at disease onset was not different between the three genotypes for the SNPs evaluated (data not shown). However, frequency analyses of risk allele in patients with early disease onset (EOPD) indicated a significant higher frequency of G-rs356219 and C-rs2736990 in patients when compared to controls (*p* = 0.040; *p* = 0.028, respectively), with OR indicating an increased risk of 1.82 (95%IC: 1.05–3.14) and 1.88 (95%IC: 1.07–3.29), respectively (**Table 4**).

**Table 4:** Comparisons of risk allele frequencies between case and control with non-motor clinical outcomes.

Outcomes	rs2583988			rs356219			rs2736990		
	T allele (%)	OR (95%CI)	*p*	G allele (%)	OR (95%CI)	*p*	C allele (%)	OR (95%CI)	*p*
**Age at onset**									
EOPD	17 (23.0)	1.24 (0.64–2.36)	0.503	46 (62.0)	1.82 (1.05–3.14)	**0.040**	50 (68.0)	1.88 (1.07–3.29)	**0.028**
Controls	38 (19.3)			93 (47.4)			79 (80.6)		
**Cognitive impairment**									
PD	34 (31.0)	2.39 (1.25–4.58)	**0.010**	72 (67.0)	2.22 (1.29–3.84)	**0.004**	76 (70.0)	2.21(1.26–3.85)	**0.005**
Controls	18 (16.0)			53 (47.0)			58 (52.0)		
**Anxiety**									
PD	31 (23.0)	1.29 (0.62–2.66)	0.591	81 (60.0)	1.47 (0.82–2.63)	0.235	89 (65.0)	1.63 (0.55–4.85)	0.398
Controls	13 (19.0)			55 (40.0)			38 (54.0)		
**Depression**									
PD	34 (24.0)	1.31 (0.61–2.81)	0.574	80 (57.0)	1.24 (0.66–2.31)	0.527	89 (64.0)	1.59 (0.88–2.87)	0.132
Controls	11 (20.0)			29 (52.0)			32 (57.0)		

*OR, odds ratio; CI, confidence interval; *p*, significance level; EOPD, early onset Parkinson’s disease; PD, Parkinson’s disease*.

The frequencies of risk alleles in cases and control groups considering only the subjects that presented cognitive impairment, anxiety and depression by clinical assessment were described in **Table 4**. There were no differences in the frequency of risks alleles between groups considering presence of depression and anxiety. However, the risk alleles T-rs2583988, G-rs356219, and C-2736990 had higher frequencies in patients (31, 67, and 70%, respectively) than controls (16, 47, and 52%) with cognitive impairments (ORs = 2.21 to 2.39). When patients were stratified by genotypes, Kruskal-Wallis test detected no differences in the scores of disease progression (HY stage), daily living activities (UPDRS-II score) or motor assessment (UPDRS-III score) between genotypes in each SNP and assessment (data not shown).

Analyses restricted to patients were performed to investigate whether risk genotypes influenced clinical outcomes (cognitive impairments, anxiety and depression). **Table 5** shows the significant and marginal associations with each SNP in SNCA by binary logistic regression. There was no significant association with motor impairment. Both rs356219 heterozygotes (OR = 4.74, 95% CI: 1.27–17.75, *p* < 0.05) and homozygotes (OR = 5.74, 95% CI: 1.42-23.21, *p* < 0.05) had significantly increased risk for cognitive impairment, while the CT-rs2736990 (OR = 3.87, 95% IC: 0.97–15.36, *p* = 0.054) and CC-rs2736990 (OR = 3.84, 95% IC: 0.96–15.30, *p* = 0.056) genotypes presented a trend toward to increased risk of the same outcome. No association of rs2583988 genotypes were found for this outcome. Conversely, TT-rs2583988 genotype significantly reduced the risk of depression (OR = 0.21, 95% CI: 0.04–0.97, *p* = 0.046) and had a marginal significance for reduced risk of anxiety (OR = 0.24, 95% CI: 0.05–1.07, *p* = 0.061). There was no significant association for rs2583988 heterozygote genotype and anxiety or depression. The SNPs rs356219 and rs2736990 had no association with these outcomes.

**Table 5:** Binary logistic regression for risk genotypes predicting cognitive, anxiety, and depression in PD patients.

	*B*	*SE*	Wald	*df*	OR (95%CI)	*p*
**Cognitive impairment**						
**rs2736990**						
CT	1.355	0.703	3.714	1	3.87 (0.97–15.36)	0.054
CC	1.346	0.705	3.641	1	3.84 (0.96–15.30)	0.056
Constant	–2.451	1.409	3.025	1		0.089
**rs356219**						
GA	1.558	0.673	5.363	1	4.74 (1.27–17.75)	**0.021**
GG	1.748	0.713	6.016	1	5.74 (1.42–23.21)	**0.014**
Constant	–2.668	1.407	3.598	1		0.058
						
**Anxiety**						
**rs2583988**						
CT	–0.490	0.515	0.905	1	0.61 (0.22–1.68)	0.341
TT	–1.427	0.763	3.501	1	0.24 (0.05–1.07)	0.061
Constant	3.364	1.474	5.212	1		0.022
						
**Depression**						
**rs2583988**						
CT	0.106	0.540	0.039	1	1.12 (0.38–3.20)	0.844
TT	–1.520	0.762	3.983	1	0.21 (0.04–0.97)	**0.046**
Constant	0.162	1.363	0.014	1		0.905

*B, regression coefficient; SE, standard error; df, degrees of freedom; OR, odds ratio; CI, confidence interval; *p*, significance level. Significant *p*-values are indicated in bold. Only significant and marginally significant associations are shown*.

The best logistic regression models (with case or control as dependent variable) performed to investigate genotypes, clinical aspects and environmental factors in prediction of disease are represented in **Table 6**. TT-rs2583988 (OR = 16.25, 95%IC: 1.72–152.97, *p* = 0.015) and CC-rs2736990 (OR = 2.37, 95%IC: 1.10–5.03, *p* = 0.026) risk genotypes were associated with increased PD risk. There was no significant improvement in the fit of the model by addition of rs356219 and rs11931074. Higher scores in BDI (OR = 1.16, 95%IC: 1.10–1.22, *p* < 0.001) and presence of cognitive impairment (OR 2.27, 95%IC: 1.03–4.98, *p* = 0.040) were directly associated with increased risk of disease. Conversely, practice of cognitive activity (OR = 0.37, 95%IC: 0.16–0.84, *p* = 0.019) and smoking habits (OR = 0.45, 95%IC: 0.21–0.97, *p* = 0.042) had a protective effect against PD.

**Table 6:** Binary logistic regression model for SNPs genotypes, clinical variables, and environmental factors predicting PD.

Variables	*B*	*SE*	Wald	*df*	OR (95% CI)	*p*
**TT-rs2583988**	2.788	1.144	5.942	1	16.25 (1.72 – 152.97)	**0.015**
**CC-rs2736990**	0.866	0.389	4.955	1	2.37 (1.10 – 5.03)	**0.026**
**BDI**	0.148	0.027	29.499	1	1.16 (1.10 – 1.22)	**<0.001**
**Cognitive Impairment**	0.943	0.400	5.558	1	2.27 (1.03 – 4.98)	**0.040**
**Cognitive Activity**	-0.983	0.417	5.547	1	0.37 (0.16 – 0.84)	**0.019**
**Smoking**	-0.784	0.385	4.147	1	0.45 (0.21 – 0.97)	**0.042**
**Constant**	-0.780	1.173	0.0442	1		0.334

*B, regression coefficient; SE, standard error; df, degrees of freedom; OR, odds ratio; CI, confidence interval; *p*, significance level; BDI, Beck Depression Inventory score. Data adjusted for sex and age. Significant *p*-values are indicated in bold*.

Logistic regression analysis revealed an association with the number of risk alleles and the presence of disease (OR = 1.27, 95% CI = 1.06–1.53, *p* = 0.009), indicating a cumulative effect. Finally, haplotype analysis result in two blocks with significant association with PD. **Table 7** shows the estimated frequency of each haplotype for cases and controls when all four markers are considered. Estimated odds ratios and associated 95% CIs indicate that haplotype T-rs2583988 + G-rs356219 + C-rs2736990 + T-rs11931074 had a greater risk for PD (OR = 2.51, 95%IC: 1.37–4.58, *p* = 0.003) than CGCT haplotype (OR = 1.85, 95%IC: 1.12–3.05, *p* = 0.017).

**Table 7:** Estimated haplotype frequencies for SNPs rs2583988, rs356219, rs2736990, and rs11931074 in SNCA gene.

Haplotype^a^	Frequency in cases (%)	Frequency in controls (%)	OR (95% CI)	*p*
**CATG**	32.1	45.7	(reference)	–
**CGCT**	29.3	22.9	1.85 (1.12 – 3.05)	0.017
**TGCG**	26.3	17.5	2.51 (1.37 – 4.58)	0.003

^a^*Haplotype is defined by rs2583988– rs356219– rs2736990– rs11931074. Adjusted for sex and age*.

*OR, odds ratio; CI, confidence interval; *p*, significance level. *p*-value uncorrected for multiple testing*.

## Discussion

The present study investigated the association between four variants of the SNCA gene and clinical outcomes in Parkinson’s disease patients compared to controls in a Brazilian sample. The risk alleles and the risk genotypes were significantly more frequent in cases than in controls and the PD risk associations for SNPs rs2583988, rs356219, and rs2736990 were confirmed for the homozygote genotype. Furthermore, rs356219 and rs2736990 demonstrated a similar frequency in cases, corroborating the positive correlation for the pairs rs356219 and rs2736990. Carriers of risk allele T-rs2583988, G-rs356219, and C-rs2736990 were significantly more frequent in cases than in controls with cognitive impairments. Regression analysis suggested associations between risk alleles and clinical outcomes and the contribution of environmental factors for PD risk.

Parkinson’s disease is a neurodegenerative, chronic, and progressive disease characterized by the presence of motor (tremor, bradykinesia, rigidity, and postural instability) and non-motor (mood disturbs, cognitive alterations, sleep, and autonomic dysfunctions) symptoms (Lauterbach, 2004; Wirdefeldt et al., 2011). Studies show higher prevalence of cognitive deficits and depression in PD (Reijnders et al., 2009; Kehagia et al., 2010) and there is a clinical association between the two kinds of symptoms (Chagas et al., 2014). Our PD sample presented moderate motor impairment, higher prevalence of anxiety and depression symptoms and a similar frequency of cognitive impairment compared to control group. Cognitive impairment is a usual finding in elderly population (Ward et al., 2012) which can explain the higher frequency of this outcome in controls, and also highlight a difficulty in identifying typical cognitive alterations of PD. Furthermore, we observed an effect of educational levels on our results. The patients with lower education presented a larger degree of cognitive impairment than controls with this same educational level. In a longitudinal study, Hindle et al. (2015) demonstrated that PD patients with a higher cognitive reserve had a better performance in cognitive tests. In addition, educational experiences are essential to attenuate age-related cognitive decline, and a major protective factor in dementia (Stern, 2009). Similarly, the practice of cognitive activities was associated with decreased risk for PD in our regression model.

Environmental factors have been related with PD in epidemiologic researches (Lau and Breteler, 2006; Wirdefeldt et al., 2011). Toxins as MPTP, herbicide paraquat and pesticide rotenone are selective complex I inhibitor and induce neuronal degeneration demonstrated *in vivo* (Betarbet et al., 2000; Lima et al., 2012) and *in vitro* (Chun et al., 2001; Uversky et al., 2002; Giordano et al., 2012) studies. Studies on the association between environmental toxins and the risk for PD present inconsistent results when rural living, pesticide use and well-water consumption are assessed (Allam et al., 2005; Firestone et al., 2005). In contrast, habits as cigarette smoking (Allam et al., 2004; Li et al., 2015) and caffeine intake (Costa et al., 2010) have been linked to protective effects, even though the mechanisms underlying these protective effects remain to be clarified. In our study, the frequencies of exposure to environmental factors were similar in cases and controls, which prevented significant associations with the disease. However, cognitive activities as reading, playing cards, board games and crossword puzzles were more frequent in controls, which suggests a protective role. To our knowledge, history of cognitive stimulation through leisure cognitive activities as a protective factor to PD has not been previously described in the literature. In this respect, the association of cognitive activities and a possible decreased risk of PD described in the present study is a new finding. However, this conclusion is limited because our protocol did not allow a precise description of type, frequency, intensity, and other aspects of the self-reported cognitive activities. It is worth mention that a recent experimental study showed that environmental stimulation facilitated motor recovery and prevented cognitive impairment in a mice model of PD (Campêlo et al., 2017). Thus, although not conclusive, the present finding encourages investigations regarding the protective role of cognitive activities in PD, possibly by prospective clinical studies.

Despite the consistent importance of environmental factors in PD etiology, most genetic association studies have not incorporated gene-environmental interactions in the researches. The interaction of smoking habits and coffee consumption and SNCA variations have been investigated, but had provided inconsistent results (De Palma et al., 1998; Gao et al., 2012; Miyake et al., 2012; Ritz et al., 2012; Trotta et al., 2012). SNPs rs2583988 and rs356219 had no association with coffee drinking and cigarette smoking when investigated in an Italian sample (Trotta et al., 2012). Similarly, the SNPs rs2736990 and rs11931074 did not demonstrate significant results in a North American study (Gao et al., 2012). However, a study in a Japanese sample reported addictive interactions between SNP rs356219 and smoking for increased risk of PD in subjects with GG- rs356219 genotype who had never smoked (Miyake et al., 2012). One of the biological mechanisms proposed to explain the protective effect of smoking is that nicotine inhibits alpha-synuclein fibrillation and stabilizes soluble oligomeric forms (Hong et al., 2009). In our sample, the logistic regression model including cognitive activities and smoking habits within TT-rs2583988 and CC-rs2736990 genotypes revealed a protective effect for PD. Taking together, these results suggest that nicotine might neutralize the detrimental effect of the risk-associated genotypes of rs2583988 and rs27366990, and a higher cognitive stimulation might be protective against PD in individuals with the risk genotypes.

Genetic data supports the role of alpha-synuclein in the pathogenic process of PD. Duplications and triplications of SNCA and a higher production of alpha-synuclein correlate with disease severity (Chartier-Harlin et al., 2004; Ibáñez et al., 2004). However, these mutations of SNCA are rare and the role of common variants are investigated as modifiers of PD susceptibility. Case-control studies have linked SNCA to sporadic PD susceptibility using SNPs analysis. Two major linkage disequilibrium blocks in SNCA gene had been proposed (Mueller et al., 2005; Myhre et al., 2008): a 5′ block that extends to promoter-enhancer region to exon 4 and a 3′ block that comprises intron, 3′ untranslated region, and the 3′ end region of the gene. Associations between SNPs rs2583988 in 5′ region (Pals et al., 2004; Winkler et al., 2007; Heckman et al., 2012; Trotta et al., 2012), rs2736990 in intron 4 (Mata et al., 2011; Heckman et al., 2012; Miyake et al., 2012; Alieva et al., 2013; Guo et al., 2014; Davila-Ortiz de Montellano et al., 2016), rs356219 (Lazzarini et al., 1994; Mata et al., 2010, 2011; Botta-Orfila et al., 2011; Wider et al., 2011; Trotta et al., 2012; Brockmann et al., 2013; Emelyanov et al., 2013), and rs11931074 (Gao et al., 2012; Wu-Chou et al., 2013) in 3′ end were demonstrated by recent studies. A meta-analysis confirmed the risk association of rs2583988, rs356219, and rs11931074 variants and PD susceptibility, performed in dominant and recessive genetic models (Han et al., 2015).

Of notice, the majority of these studies were performed in Caucasian and Asian populations. Data from Latin American populations are quite recent (Davila-Ortiz de Montellano et al., 2016; García et al., 2016), and show associations for rs3857059, rs356220, rs356203, rs7684318, and rs2736990 variants and PD in Mexican samples. Further, no prior study investigated these associations in Brazilians, or even in a South American population. Our findings were in agreement with the literature, indicating a higher PD risk for homozygote genotypes in our sample for all SNPs studied, except for rs11931074 that had no significant association with the disease. The Brazilian population is one of the most heterogeneous populations in the world and such ethnical heterogeneity creates a particular background. Variations in our findings in comparison to other studies can be explained by differences in the genetic backgrounds (Dahodwala et al., 2009). Although this feature can be a limitation due to the lack of a straight biological category, several countries in Latin America and worldwide have a multiethnic profile, which reinforces the need of genetic association studies in heterogeneous populations.

Genetic polymorphisms may contribute to specific disease characteristics and play an important role in phenotypic diversity of PD. Age at onset of PD is a predictor of progression and mortality (Wirdefeldt et al., 2011) and has been associated with multiple SNPs in several studies (Rajput et al., 2009; Yu et al., 2010; Botta-Orfila et al., 2012; Brockmann et al., 2013; Pan et al., 2013; Cardo et al., 2014; Huang et al., 2015). We found a significantly higher frequency of the G-rs356219 and C-rs2736990 risk alleles in patients with earlier onset compared to controls. Similarly, case-control studies in Germanic (Brockmann et al., 2013) and Chinese (Pan et al., 2012) samples showed that G-rs356219 and C-rs2736990 alleles significantly contributed to earlier age onset. In contrast, a lack of association was observed in Italians (Trotta et al., 2012) and North Americans (Mata et al., 2011; Heckman et al., 2012), which suggest the participation of other modifying factors in age onset across different populations. Therefore, the contributions of these polymorphisms to age onset remain unclear. We speculate that increased expression of alpha-synuclein protein may result in an early manifestation of PD symptoms.

Studies have investigated the functional effects of the different SNCA SNPs on gene expression in brain tissues and protein levels in blood samples, but the results were inconsistent (Fuchs et al., 2008; Linnertz et al., 2009; Mccarthy et al., 2011; Alieva et al., 2013; Cardo et al., 2014). For example, a study with a transgenic mouse model demonstrated that REP1 variants in 5′ region affect the regulation of transcriptional activity (Cronin et al., 2009). Human studies demonstrated reduction of SNCA-mRNA levels in brain tissues and protein levels in blood in the absence of REP1 risk allele (Fuchs et al., 2008; Linnertz et al., 2009). Despite some evidence of correlations between rs2583988 and REP1 variants (Pals et al., 2004; Winkler et al., 2007; Myhre et al., 2008), no significant associations of rs2583988 were found for assessment of blood protein levels (Fuchs et al., 2008) or SNCA-mRNA (Fuchs et al., 2008; Linnertz et al., 2009; Alieva et al., 2013).

For the intronic SNP rs2736990, an investigation of gene expression revealed a trend toward lower levels of SNCA-mRNA in blood samples of PD patients (Alieva et al., 2013), but in healthy subjects the T allele was correlated with higher levels of the isoform SNCA112-mRNA in frontal cortex tissue samples.

SNPs rs356219 and rs11931074 are part of 3′ block and variants in this region may affect post-transcriptional regulation factors, such as biding sites of mRNA, and impair RNA processing or stability and gene or protein expression (Linnertz et al., 2009; Venda et al., 2010; Elbaz et al., 2011; Mccarthy et al., 2011). For rs356219, the heterozygote genotype was correlated with higher levels of mRNA in substantia nigra of PD cases (Fuchs et al., 2008). Further, G allele carriers presented higher levels of the SNCA112-mRNA isoform in frontal cortex (Mccarthy et al., 2011). However, a divergent result was found by Linnertz et al. (2009) that showed a correlation between higher levels of SNCA-mRNA and the protective allele A.

As mentioned, literature supports that polymorphisms in the SNCA gene increase genetic susceptibility to sporadic PD and suggests an increased expression of alpha-synuclein. Despite disease severity (rapid cognitive decline, severe non-motor symptoms, more widespread neurodegeneration and faster disease progression) is related to increased alpha-synuclein expression in familial PD (Venda et al., 2010), there is a lack of evidence regarding the SNCA variants’ influence on specific clinical outcomes. Previous studies that assessed associations between polymorphisms and motor impairment and disease progression (Ritz et al., 2012; Markopoulou et al., 2014; Cheng et al., 2016; Davis et al., 2016; Wang et al., 2016), cognitive functions (Goris et al., 2007; Guo et al., 2014; Markopoulou et al., 2014; Chen W. et al., 2015; Wang et al., 2016) and presence or absence of anxiety and depression (Guo et al., 2014; Chen W. et al., 2015; Cheng et al., 2016; Dan et al., 2016) present poorly consistent results.

In our study, no associations were observed regarding the distribution of risk allele and presence of depression or anxiety. Nevertheless, here we indicate that the TT- rs2583988 genotype has a protective effect against these outcomes in patients. In contrast, a study with a Chinese sample found a decreased risk of depression in carriers of REP1 risk homozygote genotype and a correlation with UPDRS part II score, motor fluctuation and female sex in prediction of PD depression (Dan et al., 2016).

Concerning cognitive aspects, T-rs2583988, C-rs2736990, and G-rs356219 risk alleles were more frequent in cases than controls with cognitive impairment, indicating a greater risk of this outcome in PD. Furthermore, we found an association of cognitive impairment with risk of PD in the logistic regression model. An unexpected found for REP1 genotypes was described in a longitudinal North American study: PD cases with higher REP1 scores were associated with better motor function and reduced risk of cognitive impairments (Markopoulou et al., 2014). These data highlight a possible dual effect or time-dependent role for SNCA variants.

Regarding motor aspects, a cohort study with American patients (Ritz et al., 2012) demonstrated an increased risk of faster decline of motor function in carriers of the REP1 263bp promoter variant and G-rs356165 allele. In our transversal study, we did not found correlations between genotypes and severity of motor symptoms in the patients.

Our study had some limitations. Environmental data were self-reported, which could provide a misclassification. Further, the MMSE used to assess cognitive impairment may not be the limited number of patients, the power analysis indicated a moderate statistical power. Irrespective, it would be interesting to expand the sample to reinforce the results. Indeed, the few conflicting results may be explained by the small sample size and differences in genetic background among different ethnic populations, as mentioned.

## Conclusion

This study confirms the association between PD and SNCA SNPs and haplotypes in a Brazilian population. Further, the data provide evidence that the SNCA variants are associated with increased risk of cognitive impairment in PD patients. Therefore, our results encourage the investigation of associations between genetic variants in SNCA and specific clinical outcomes. Phenotypic studies and functional assays, with a large sample and different ethnicities should be performed to confirm and specify the nature of the associations.

## Ethics Statement

This study was carried out in accordance with the recommendations of Comissão Nacional de Ética em Pesquisa (CONEP), Brazil, with written informed consent from all subjects. All subjects gave written informed consent in accordance with the Declaration of Helsinki. The protocol was approved by the ethical committee of Onofre Lopes University Hospital (protocol number 04261012.5.1001.5292).

## Author Contributions

CC and RS designed the research and wrote the paper. CC, FC, LO, AS-N, and PT collected the data. CC, DdS, and TdA conducted molecular assays. CC. analyzed the data. JS, GI, AR, and CdO contributed with theoretical support and data analysis.

## Conflict of Interest Statement

The authors declare that the research was conducted in the absence of any commercial or financial relationships that could be construed as a potential conflict of interest.

## Footnotes

1 http://bioinfo.iconcologia.net/SNPstats
